# Motor skills in relation to body-mass index, physical activity, TV-watching, and socioeconomic status in German four-to-17-year-old children

**DOI:** 10.1371/journal.pone.0251738

**Published:** 2021-05-17

**Authors:** Siegfried Möller, Tanja Poulain, Antje Körner, Christof Meigen, Anne Jurkutat, Mandy Vogel, Sven Wessela, Andreas Hiemisch, Nico Grafe, Wieland Kiess

**Affiliations:** 1 Department of Women and Child Health, University Hospital for Children and Adolescents and Center for Pediatric Research, Leipzig University, Leipzig, Saxony, Germany; 2 LIFE Leipzig Research Center for Civilization Diseases, Leipzig University, Leipzig, Saxony, Germany; The Education University of Hong Kong, HONG KONG

## Abstract

**Background:**

The present study describes motor skills in a large sample of German children and adolescents and investigates associations with age, gender, body-mass index, physical activity, television time, and socioeconomic status.

**Methods:**

2,106 children (1076 boys, 1030 girls) aged 4 to 17 years performed five different motor tests for strength (pushups, standing long jump), coordination (backward balancing, jumping side-to-side) and flexibility (forward bend) within the framework of the LIFE Child study (Leipzig, Germany). Anthropometric parameters were assessed through standardized measurement. Data on physical activity, television time, and socioeconomic status were collected via questionnaires. Linear regression analyses were applied to assess relations.

**Results:**

Strength and coordination performance were higher in older than in younger children. While boys showed a higher performance in strengths tests than girls, girls performed better in flexibility and coordination during precision tasks (backward balancing). In terms of coordination under time constraint (jumping side-to-side), both genders produced similar results. Lower body-mass index, higher physical activity, and higher socioeconomic status were significantly related to better motor skills. Longer television times were significantly associated with lower performance in long jump.

**Conclusions:**

The present findings are similar to data collected at the beginning of the century, indicating that motor skills have hardly changed in recent years. The findings furthermore suggest that children from lower social strata, children with higher body weight, and children who move little have a higher risk of developing insufficient motor skills and should therefore be given special support.

## Introduction

Physical activity and motor performance promote mobility, coordination, posture, and balance and are important building blocks for healthy development and wellbeing. Against this background, it is worrying that the physical activity of children and adolescents in Germany [1, 2] as well as in other countries [3] is insufficient and that the (aerobic) fitness of children and adolescents has decreased in recent years [4, 5]. At the same time, sedentary behavior has increased rapidly, mainly due to increased media use [6, 7], and proportions of overweight and obesity have stabilized on a high level [8].

The present study aims to describe motor skills in a large sample of four- to 17-year-old German girls and boys. To provide maximum comparability, motor skills were assessed using five tests of the Motorik-Modul, a test developed to evaluate motor skills in German youth [9]. The five tests were chosen to assess different aspects of motor competence: strength, coordination, and flexibility. Data collected between 2003 and 2006 in a representative sample of German 4- to 17-year-old children demonstrated higher performances in older versus younger children in all tests except flexibility [10]. Whereas male children performed better in strength tests (at least from 14 years on), girls performed better in tests of coordination and flexibility [10]. Since these data were collected more than 10 years ago, it is important to provide more up-to-date data on the motor skills of children and adolescents. Overall, we expected to find the same age and gender differences as Woll et al. [10]. However, a comparison of the findings might reveal some differences between motoric skills of the current and the previous generation of youth.

Another aim of the present study was to assess associations between motor skills in children and BMI, physical activity (PA), sedentary behavior (TV time), and socioeconomic status (SES). Previous studies suggest relations between motor skills and lower BMI [11–14]. Possible reasons for this association are that children with higher BMI are more likely to suffer from movement-restricting orthopedic problems and have greater difficulty in moving or coordinating their bodies [11, 15]. PA, in turn, has been associated with better motor skills [16–18]. This association might suggest that less activity leads to poorer motor performance. At the same time, children with poorer motor skills might experience subjective limitations and inferiority when engaging in PA and might, therefore, reduce their PA [18–21]. Regarding SES, it is well documented that a lower SES of the family is associated with a higher BMI, less PA, and more sedentary behavior [22, 23], but also with lower motor skills [24]. Overall, these findings suggest a complex interplay between BMI, PA, SES, and motor skills. Therefore, the present study investigated these parameters in the same sample of children and adolescents and included them in the same statistical model. This strategy made it possible to assess independent associations with motor skills. Based on previous findings, we expected to find associations of better motor skills with lower BMI, higher PA, less sedentary behavior, and higher SES.

## Methods

### Participants

The present study was realized within the LIFE Child study, a childhood cohort study investigating the development of children from infancy into adulthood with a particular focus on lifestyle diseases [25, 26]. All children interested in the LIFE Child study are invited to participate. They are recruited via advertisement at different institutions, e.g., schools or health centers, and mainly come from the city or surrounding areas of Leipzig, a city with 600.000 inhabitants situated in Eastern Germany. Only children with chronic, chromosomal, or syndrome diseases are excluded from participation [25, 26].

**Fig 1:**
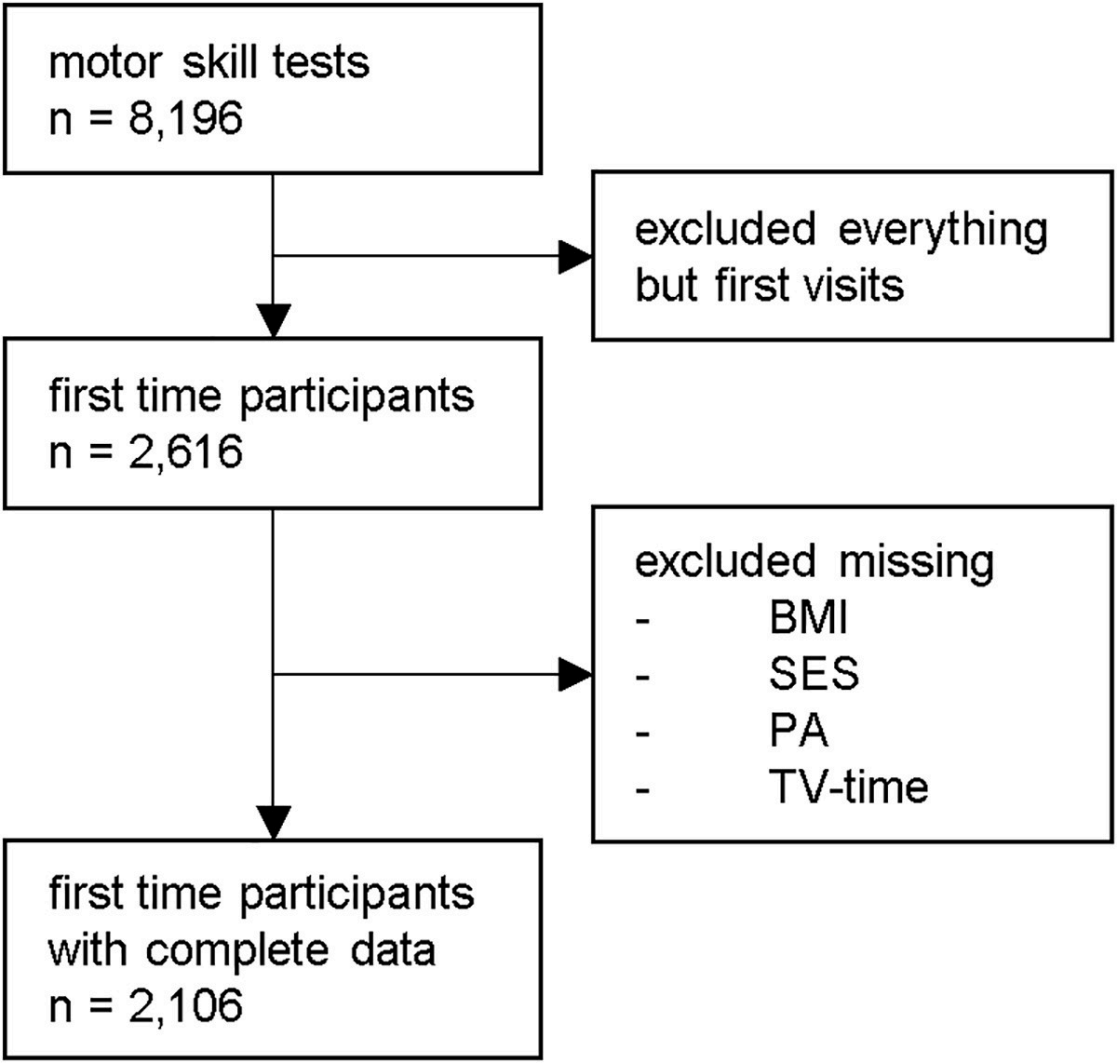
The final sample. BMI = Body-Mass-Index, SES = socioeconomic status, PA = physical activity.

The data used for the present project were collected between 2011 and 2017. All participants aged between 4 and 17 years for whom information on motor skills, BMI, PA, TV time, and SES were available were included in the analysis. In the case of multiple study visits per child, only the first visit was considered (Fig 1). All parents gave informed consent, and, from the age of 12 years, the children themselves also gave written consent.

The study was designed in accordance with the Declaration of Helsinki [27] and was approved by the Ethics Committee of the Medical Faculty of the University of Leipzig (chairperson: Prof. Ortrun Riha, Reg. No. 264/10-ek, date of approval: October 7, 2010).

### Measures

#### Motor skills test

The motor skills test consists of five exercises from the Motorik-Modul (MoMo) of the German Health Interview and Examination Survey for Children and Adolescents (KiGGS) [10]. The Motorik-Modul is a reliable and valid test to assess physical fitness in 4- to 17-year-old children and adolescents [10]. It was created in a modular way, allowing researchers to either adapt the whole test battery, single tests or a sample of various test items. We selected five tests that represent different aspects of gross motor skills and, therefore, allow an evaluation of multiple different aspects of motor performance.

Standing long jump was used to assess locomotive strength. Participants were asked to jump as far as possible with their feet closed. The better of two attempts was counted. Attempts were declared invalid if participants stepped over the line during takeoff or moved their feet after landing. To assess strength endurance of the upper extremity, participants were asked to perform the pushup test, in which they were asked to do as many pushups as possible in 40 seconds. In terms of coordination, backward balancing evaluates coordination during dynamic precision tasks, while jumping side-to-side estimates coordination under time constraint. To do the backward balancing tests, participants were asked to balance backwards on three beams of different widths. They had two attempts per beam. The numbers of steps were counted (up to eight steps per attempt) and added up to a final score. An attempt was terminated if a participant’s foot or hand touched the ground or wall. In the jumping side-to-side test, participants were asked to jump side to side from one rectangle painted on the ground to an adjacent one for 15 seconds. After two attempts, both jump counts were averaged for a final score. Finally, forward bend evaluates flexibility of the trunk. Participants stood on a bench and were asked to reach downwards with their hands while keeping their knees in full extension. The distance between their fingertips and the bench was noted, with positive values marking that the bench level was exceeded. The better of two attempts was counted. All tasks were performed as described by Woll et. al [10] in a sports room at the Leipzig Research Center for Civilization Diseases. To ensure a standardized procedure, all study assistants were trained and regularly certified.

#### Covariates

In this analysis, age, gender, PA, TV time, SES, and age- and gender-specific standard deviation scores of children’s BMI (BMI-SDS) were considered as covariates. Information was collected by questionnaires and examinations. For participants aged 4 to 10 years, questionnaires on PA and TV time (see S1 Table) were completed by parents. Participants aged 11 years and older completed questionnaires on their own.

For the assessment of children’s BMI, weight and height were measured by trained research assistants, following a standardized procedure. BMI was transformed to standard deviation scores (BMI-SDS) based on German age- and gender-specific references [28]. For further analysis, participants with a BMI equal to the 90^th^ percentile or greater were considered overweight/obese, according to guidelines of the Arbeitsgemeinschaft Adipositas (AGA) [29].

To assess PA, participants were asked to indicate the frequency of activities in sports clubs. Five answer categories could be chosen: “never”, “less often than once per week”, “1–2 times per week”, “3–5 times per week”, “almost every day”. This question was part of a physical activity questionnaire originally designed for the use in the KiGGs study [30]. For further analysis, children were assigned to either the “low/normal” (the lower three categories) or the “high” (the higher two categories) PA group. The distinction between low/normal and high PA was based on a median split. This approach was already applied in another study [31].

For the assessment of TV time, participants were asked to indicate how many hours per day they usually spend watching TV. Five answer categories could be chosen: “never”, “30 minutes”, “1 to 2 hours”, “3 to 4 hours”, “more than 4 hours.” This question was part of a media use questionnaire originally designed for the KiGGs study [32]. According to the response, TV time was categorized as either “short/normal” or “long”. For participants aged 4 to 10 years, TV times of at least 1 hour were considered “long”. For children aged 11 years or older, TV times of more than 2 hours were considered “long”. These cut-offs were chosen based on current screen-time recommendations for children and adolescents [33, 34].

Participants’ SES was assessed as a combination of parents’ education (graduation and professional education), their occupational status, and their monthly household equivalent income. The final SES score can range between 3 and 21, with higher scores indicating higher SES [35]. Following cut-offs created based on a representative survey on SES in German families [35], the SES score can be categorized as either low, middle, or high.

### Statistical analysis

All statistical analyses were performed using the software R (version 3.5.1). To evaluate possible associations of motor skills with age and sex, we performed multiple linear regression analyses with the raw values in the five motor skills tasks as dependent variables and age and sex as independent variables. To assess associations between motor skills and the other covariates, we applied another series of multiple linear regression analyses. Age- and gender-standardized scores (SDS) of the motor skills tasks were included as dependent variables, BMI, SES (as continuous measure), PA (“low/normal” versus “high”), and TV time (“short/normal” versus “long”) were included as independent variables, and age and sex were included as covariates. The age- and sex-specific scores were calculated based on all motoric tests performed in the LIFE Child study. Generalized additive models for location, scale and shape [36] were applied.

Each model was checked for collinearity between the independent variables. The variance inflation factor (VIF) of each variable did never exceed the value of five, indicating that the inclusion of the variables in the same model did not reduce model quality. In addition, each statistical model was checked for interactions between the independent variables and age and gender. An interaction was included in the final model if the interaction term reached significance (p < 0.05) and did not cause an inflation of variance (VIF < 5). All p-values were adjusted for multiple testing according to the False Discovery Rate method.

## Results

### Study population

In total, 2106 children (1030 girls, 1076 boys; mean age = 10.06 years, age range = 4.38–17.94 years) met the inclusion criteria. Table 1 shows the distribution of age, gender, SES, and BMI among participants. Distributions of low/normal PA vs. high PA as well as short/normal TV time vs. long TV time are shown in Table 2, stratified by age group (4- to 10-year-olds versus 11- to 17-year-olds). As can be seen, the population was characterized by a rather high SES (proportion of high SES > 40%). Overweight/obesity was present in 20% of all participants. While the proportions of high levels of PA were lower in younger (5- to 10-year-old) than in older (11- to 17-year-old) children (12% versus 23%), the proportion of long TV times was higher in younger than in older children (37% versus 22%). However, it is important to note the different categorization algorithms for TV times in younger (all TV times > 1 hour/day considered as high) and older children (TV times > 2 hours/day considered as high).

**Table 1:** Characteristics of the study population.

	Sample size N = 2106
Gender (n (%)):	
male	1076 (51.1%)
female	1030 (48.9%)
Age in years (mean (standard deviation))	10.06 (3.33)
SES^1^ (n (%)):	
low	240 (11.4%)
middle	971 (46.1%)
high	895 (42.5%)
SDS-BMI^2^ (n (%)):	
< 90^th^ percentile	1680 (79.8%)
> = 90^th^ percentile	426 (20.2%)
Number of performed motoric tasks	
Long jump	1896 (90.0%)
Pushup	1915 (90.9%)
Side to side	2025 (96.2%)
Balancing	2072 (98.4%)
Forward bend	2072 (98.4%)

^1^ Categorization according to Lampert et al. [33]

^2^ in percentiles, according to Kromeyer-Hauschild et al. [28], SES = socioeconomic status, SDS-BMI = standard deviation score body-mass index.

**Table 2:** Physical activity and television time amongst the study population.

	4- to 10-year-olds*	11- to 17-year-olds**
	N = 1304	N = 825
Organized physical activity:		
low/normal (< = 2 / week)	1130 (88.2%)	635 (77.0%)
high (> 2 / week)	151 (11.8%)	190 (23.0%)
Television time***:		
short/normal	804 (62.8%)	643 (77.9%)
long	477 (37.2%)	182 (22.1%)

* Parental report

** Child report

*** Cut-offs for short/normal TV times were <1h/d for 4- to 10-year-olds and <2h/d for 11- to 17-year-olds.

p-values were adjusted for multiple testing according to the False Discovery Rate method.

The motor skills test was performed by each participant. However, some participants failed to perform specific tasks correctly, resulting in different numbers of tests that could be analyzed (see Table 1).

### Motor skills in relation to child age and gender

**Fig 2:**
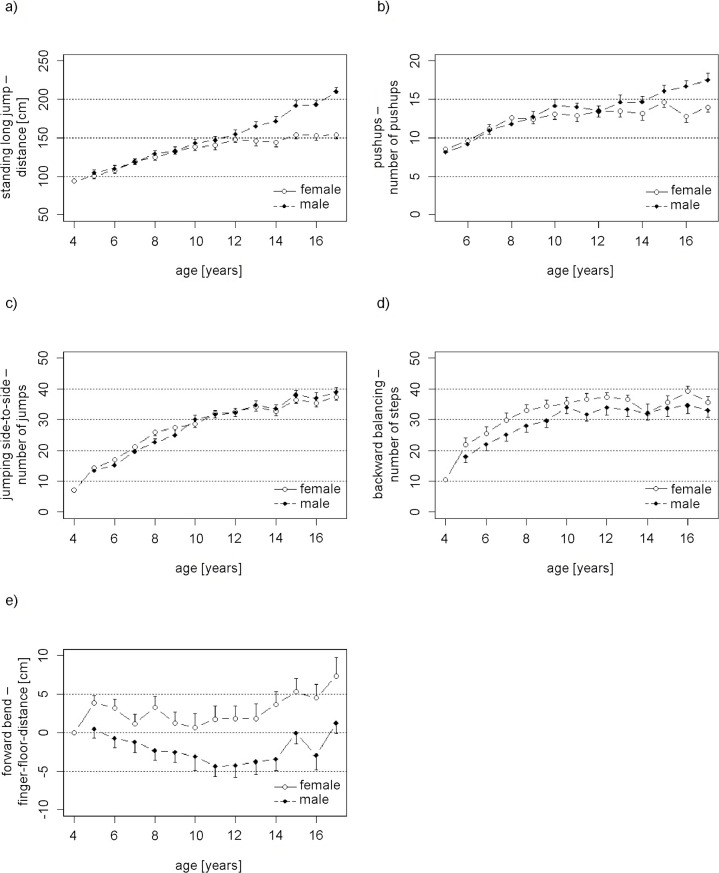
Raw scores in the different tasks of the motoric test by age and gender. (a) Best distance of two tries doing a standing long jump. (b) Number of pushups during 40s. (c) Mean number of side-to-side jumps performed in a 15s interval. (d) Number of steps taken on six tries balancing backwards on three beams of different widths. (e) Best distance (cm) between fingertips and bench reached in two tries (negative numbers indicate bench level was not reached, positive numbers indicate bench level was exceeded). Error bars indicate 95% confidence intervals.

The associations between the results of the different motor skills tasks (raw scores) and age and gender are shown in Table 3 and Fig 2. For the strength tests standing long jump and pushup test, the analyses revealed a significantly higher performance in older than in younger children. Furthermore, performance was significantly lower in girls than boys.

**Table 3:** Association of motor skills with age and gender.

	Long jump		Pushup		Side to side		Balancing		Forward bend	
	b (CI)	p	b (CI)	p	b (CI)	p	b (CI)	p	b (CI)	p
age	6.42	<0.001	0.57	<0.001	2.26	<0.001	1.38	<0.001	-0.08	0.16
	(6.08		(0.52		(2.18		(1.24		(-0.18	
	– 6.76)		– 0.62)		– 2.35)		– 1.52)		– 0.01)	
gender*	-9.84	<0.001	-0.54	0.003	0.46	0.19	3.27	<0.001	4.88	<0.001
	(-12.03		(-0.87		(-0.11		(2.35		(4.24	
	to -7.64)		to -0.22)		– 1.03)		– 4.19)		– 5.51)	

Age and gender were included simultaneously (multivariate regression analyses).

b = non-standardized regression coefficients. CI = 95% confidence interval.

* reference = male.

p-values were adjusted for multiple testing according to the False Discovery Rate method.

For the two tests of coordination (jumping side to side and backward balancing), analyses showed significant increases with increasing age. A significant gender difference was only observed for backward balancing, with girls performing better than boys.

The test of flexibility (forward bend) was the only test for which no significant association with age was found. As for backward balancing, girls performed significantly better than boys.

The analyses revealed no significant interactions between age and gender (all p > 0.05), indicating that the associations between the raw scores and age did not differ between girls and boys.

### Motor skills in relation to BMI, PA, TV time, and SES

The motor skills (sex- and age adjusted scores) of children with overweight/obesity versus normal weight, with high versus low/normal levels of PA, with long versus short/normal TV times, and with low versus middle versus high SES are shown in S1–S4 Figs. The results of the analyses on the independent associations of these factors with motor skills are shown in Table 4. For the strength tests (standing long jump and pushups), the analyses showed significant associations between higher test results and lower BMI, high PA and higher SES. Regarding SES, the mean jumping distance of participants with a low SES (SES score of 6) was estimated -0.23 SDS, compared to -0.01 SDS for middle SES (SES score of 12) and 0.22 SDS for high SES (SES score of 18). For pushups, the estimated average test result was -0.21 SDS for low SES, -0.04 SDS for middle SES, and 0.13 SDS for high SES. Additionally, better test results in the long jump test were significantly associated with low/normal TV times.

**Table 4:** Associations of motor skills with BMI, organized physical activity (PA), television time (TV), and socioeconomic status (SES): Results of multivariate regression analyses.

	Long jump		Pushup		Side to side		Balancing		Forward bend	
	b (CI)	p	b (CI)	p	b (CI)	p	b (CI)	p	b (CI)	p
BMI	-0.29	<0.001	-0.17	<0.001	-0.14	<0.001	-0.28	<0.001	-0.03	0.19
	(-0.32 to -0.39		(-0.20 to -0.13)		(-0.18 to -0.11)		(-0.32 to -0.20)		(-0.06–0.01)	
PA	0.35	<0.001	0.50	<0.001	0.41	<0.001	0.17	0.01	0.36	<0.001
	(0.24–0.46)*		(0.39–0.62)*		(0.29–0.52)		(0.05–0.29)		(0.24–0.47)*	
TV	-0.23	0.003	-0.02	0.76	0.02	0.83	-0.09	0.10	-0.08	0.17
	(-0.36 to -0.09)		(-0.12–0.07)*		(-0.12–0.17)		(-0.19 to -0.0004)		(-0.17–0.01)	
SES	0.04	<0.001	0.03	<0.001	0.04	<0.001	0.03	<0.001	0.01	0.36
	(0.03–0.05)		(0.02–0.04)		(0.03–0.06)		(0.02–0.05)		(-0.004–0.02)	

BMI (as continuous measure), PA (high versus low/normal), TV (long versus short/normal), and SES (as continuous measure) were included simultaneously (multivariate regression analyses). All associations were adjusted for age and gender.

b = non-standardized regression coefficient. CI = 95% confidence interval.

*A significant interaction with gender indicated significantly stronger associations in girls versus boys (see text for more details).

For both coordination tests (jumping side to side and backward balancing), the analysis showed significant associations with lower BMI, high PA, and higher SES. With respect to SES, the estimated average test result in jumping side to side was -0.38 SDS for children from low SES, -0.11 SDS for middle SES, and 0.15 SDS for high SES. The performance in backward balancing of children with a low SES was estimated -0.19 SDS, compared to 0.004 SDS for middle SES and 0.20 SDS for high SES.

For the flexibility test (forward bend), the analysis showed a significantly higher test result in children showing high levels of PA than in children showing low/normal levels of PA.

All associations between motor skills and the covariates were checked for interactions with age and gender. These analyses revealed five significant interactions with age, which, however, created inflations of variance (all VIF > 11) and, therefore, were excluded from the models. Regarding interactions with gender, one significant interaction was observed, and did not cause an inflation of variance (VIF = 1.8). The interaction indicated significantly stronger associations of PA with performance in forward bend in girls (b = 0.61 (95% CI 0.38–0.84)) than in boys (b = 0.19 (95% CI 0.04–0.34)).

## Discussion

The objective of this study was to document the motor skills of 4- to 17-year-old boys and girls growing up in Germany and to identify factors that are associated with motor skills. The analysis showed that relations with age and gender vary between the specific motor skills. Age was associated positively with all tasks except flexibility. While boys performed better in strength tasks, girls showed better performance in coordination with precision and in flexibility. Overall, these findings are highly similar to the results from a nationwide assessment of motor skills using the same tests 10–15 years ago [10, 37]. The only difference that emerges when comparing the present data with data from Woll et al. [10] is a slightly higher number of pushups in the present study. One possible reason for this discrepancy might be that children in Leipzig participate more frequently in organized sports, where pushups represent a frequent exercise. In our sample, 72% of boys and 72% of girls engaged in organized sports, compared to 63% and 52% in the study by Woll et al. [10].

When compared with studies that used different test items to evaluate motor skills and were performed with populations different from the one of this study, the overall findings are highly comparable. Research from many other European countries and China suggest that boys performing better than girls in various strength tests while being outperformed by their female peers in terms of flexibility is consistent in different populations [38, 39]. An American study regarding strength in children and adolescents supports the findings of this study, that sex-specific differences in strength emerge in adolescence [40] (compare Fig 2).

The analysis regarding BMI, PA, TV time, and SES showed significant associations between high levels of PA and higher performance in all tasks of the motoric test. Higher BMI and lower SES were related to lower performances in all tasks except the flexibility test. Long TV times were associated with lower performance in long jump and backward balancing.

### Association with BMI

We observed significant associations between higher BMI and lower motor skills. These associations are in line with findings of other studies performed in different parts of the world and using various test batteries to evaluate motor skills [11–14]. Our findings of significant associations between higher BMI and lower motor skills were independent of other cofactors, including PA. One possible explanation is that a higher BMI results in lower motor skills, e.g., because a higher weight makes a lot of tasks like jumping, running and lifting one’s own body weight more difficult in a biomechanical way [15]. Furthermore, children with overweight/obesity might be more frequently affected by orthopedic problems [11], which might cause pains and, therefore, reduce motoric performance. Given the cross-sectional design of the study, we cannot rule out that lower motor skills might also cause higher BMI, e.g. through reduced PA, a risk factor for overweight/obesity in children [41].

### Association with PA

We observed that children engaging less frequently in PA had lower motor skills than highly active children. This finding is in line with an Italian study and a meta-analysis of international studies, both showing that PA-intervention can improve overall gross, locomotor and object-control motor skills [17, 42]. Importantly, the associations between PA and motor skills were independent of children’s BMI, indicating that this association cannot be explained by BMI (alone). The finding strongly suggests that adequate PA is an important cornerstone for motor skills in children and adolescents [43]. At the same time, low motor skills might limit the motivation to move and, therefore, (further) reduce PA [18, 44, 45].

Interestingly, the analyses showed stronger associations between PA and performance in the forwards bend task in girls than in boys. One possible explanation is that activity in sports clubs differentiates better between physically active and inactive girls than between physically active and inactive boys. Boys’ activity might be less restricted to activity in sports clubs. Differences in the types of sports in which girls versus boys participate could also play a role.

### Association with television

Watching TV, a type of sedentary behavior, showed the weakest and, with the exception of the association with results in the long jump task, only non-significant associations with motor skills of children and adolescents. This contradicts findings of a systematic review of 232 studies with participants from 39 countries showing negative associations of TV-watching with physical and musculoskeletal fitness in most cases [46]. A possible explanation is that TV-watching in itself is not detrimental to motor skills as long as it doesn’t lead to a decrease in PA or a higher BMI.

### Association with SES

In addition to BMI, PA (and TV time), lower motor skills were also related to a lower SES. This finding is in line with a systematic review and meta-analysis of 59 studies on correlates of movement skills and motor coordination [24] and with the general observation of lower health behavior, health awareness, and health outcomes among children from poorer social backgrounds [23]. Parents from lower social levels might have less time, fewer financial possibilities, and might be less motivated to promote an active leisure style and motor skills of their children (e.g. through frequent outdoor activities within the family or involvement in sports clubs).

### Strengths and limitations

The strengths of the study are the large sample and the consideration of independent associations of numerous factors with motor skills. However, some limitations have to be mentioned. First, this is a cross-sectional study. The results therefore do not allow conclusions about the direction of effects. Other limitations are the underrepresentation of children from low social strata and the focus on children growing up in an urban area. These limitations might reduce the generalizability to the whole population of children and adolescents. Further, this study does not take into account biological maturity. There is research that suggests biological maturity has an important influence on physical fitness measures during adolescence, especially in boys [47, 48]. Part of the variations in motor skill test results might be attributed to differences in biological maturity. Finally, information on PA and TV time is based on data in questionnaires that have not been validated. It is possible that the actual physical activity and the actual TV time differ from the information provided.

## Conclusions

Overall, the current motor skills of children and adolescents from an urban area in Easter Germany are comparable with the skills of the previous generation of young people. This suggests that the motor skills of children have hardly changed in recent years. Higher BMI, lower levels of PA, lower SES, and–to a lesser extend–longer TV times are independently associated with lower motor skills. These findings suggest that children with overweight/obesity, children from lower social strata, and children who move little have an increased risk of developing insufficient motor skills. Special support should therefore be given to these groups of children (e.g. through free sports activities in and outside school).

## Supporting information

S1 Fig **Raw scores in the different tasks of the motoric test by age, weight status, and gender (boys on the left, girls on the right).** (a) best distance of two tries doing a standing long jump. (b) number of pushups during 40s. (c) mean number of side to side jumps performed in a 15s interval. (d) Number of steps taken on six tries balancing backwards on three beams of different widths. (e) best distance (cm) between fingertips and bench reached in two tries (negative numbers indicate bench level was not reached, positive numbers indicate bench level was exceeded). Error bars indicate 95% confidence intervals.
(TIF)

S2 Fig **Raw scores in the different tasks of the motoric test by age, level of PA, and gender (boys on the left, girls on the right).** (a) best distance of two tries doing a standing long jump. (b) number of pushups during 40s. (c) mean number of side to side jumps performed in a 15s interval. (d) Number of steps taken on six tries balancing backwards on three beams of different widths. (e) best distance (cm) between fingertips and bench reached in two tries (negative numbers indicate bench level was not reached, positive numbers indicate bench level was exceeded). Error bars indicate 95% confidence intervals.
(TIF)

S3 Fig **Raw scores in the different tasks of the motoric test by age, TV time, and gender (boys on the left, girls on the right).** (a) best distance of two tries doing a standing long jump. (b) number of pushups during 40s. (c) mean number of side to side jumps performed in a 15s interval. (d) Number of steps taken on six tries balancing backwards on three beams of different widths. (e) best distance (cm) between fingertips and bench reached in two tries (negative numbers indicate bench level was not reached, positive numbers indicate bench level was exceeded). Error bars indicate 95% confidence intervals.
(TIF)

S4 Fig **Raw scores in the different tasks of the motoric test by age, SES, and gender (boys on the left, girls on the right).** (a) best distance of two tries doing a standing long jump. (b) number of pushups during 40s. (c) mean number of side to side jumps performed in a 15s interval. (d) Number of steps taken on six tries balancing backwards on three beams of different widths. (e) best distance (cm) between fingertips and bench reached in two tries (negative numbers indicate bench level was not reached, positive numbers indicate bench level was exceeded). Error bars indicate 95% confidence intervals.
(TIF)

S1 Table Questions of the leisure-time-behavior questionnaires. (DOCX)

## References

[1] Schwarzfischer P, Weber M, Gruszfeld D, Socha P, Luque V, Escribano J, et al. BMI and recommended levels of physical activity in school children. BMC Public Health 2017;17(1):595. 10.1186/s12889-017-4492-4 28645324 PMC5482946

[2] Finger JD, Varnaccia G, Borrmann A, Lange C, Mensink GBM. Körperliche Aktivität von Kindern und Jugendlichen in Deutschland–Querschnittergebnisse aus KiGGS Welle 2 und Trends. Journal of Health Monitoring 2018:3(1). 10.17886/RKI-GBE-2018-006

[3] Hallal PC, Andersen LB, Bull FC, Guthold R, Haskell W, Ekelund U. Global physical activity levels: surveillance progress, pitfalls, and prospects. The Lancet 2012;380(9838):247–57. 10.1016/S0140-6736(12)60646-1 22818937

[4] Knuth AG, Hallal PC. Temporal trends in physical activity: a systematic review. J Phys Act Health 2009;6(5):548–59. 10.1123/jpah.6.5.548 19953831

[5] Tomkinson GR, Léger LA, Olds TS, Cazorla G. Secular trends in the performance of children and adolescents (1980–2000): an analysis of 55 studies of the 20m shuttle run test in 11 countries. Sports Med 2003;33(4):285–300. 10.2165/00007256-200333040-00003 12688827

[6] Auhuber L, Vogel M, Grafe N, Kiess W, Poulain T. Leisure activities of healthy children and adolescents. Int J Environ Res Public Health 2019;16(12). 10.3390/ijerph16122078 31212786 PMC6617342

[7] Bucksch J, Sigmundova D, Hamrik Z, Troped PJ, Melkevik O, Ahluwalia N, et al. International trends in adolescent screen-time behaviors from 2002 to 2010. J Adolesc Health 2016;58(4):417–25. 10.1016/j.jadohealth.2015.11.014 26827267

[8] Keß A, Spielau U, Beger C, Gausche R, Vogel M, Lipek T, et al. Further stabilization and even decrease in the prevalence rates of overweight and obesity in German children and adolescents from 2005 to 2015: a cross-sectional and trend analysis. Public Health Nutr 2017;20(17):3075–83. 10.1017/S1368980017002257 28931448 PMC10261442

[9] Woll A, Albrecht C, Worth A. Motorik-Modul (MoMo)–das Modul zur Erfassung der motorischen Leistungsfähigkeit und der körperlich-sportlichen Aktivität in KiGGS Welle 2. Journal of Health Monitoring 2017;2(S3):66–73. 10.17886/RKI-GBE-2017-1

[10] Woll A, Kurth B-M, Opper E, Worth A, Bös K. The ‘Motorik-Modul’ (MoMo): physical fitness and physical activity in German children and adolescents. Eur J Pediatr 2011;170(9):1129–42. 10.1007/s00431-010-1391-4 21318230

[11] Okely AD, Booth ML, Chey T. Relationships between body composition and fundamental movement skills among children and adolescents. Res Q Exerc Sport 2004;75(3):238–47. 10.1080/02701367.2004.10609157 15487288

[12] Milne N, Leong GM, Hing W. The relationship between children’s motor proficiency and health-related fitness. J Paediatr Child Health 2016;52(8):825–31. 10.1111/jpc.13236 27439732

[13] Goulardins JB, Rigoli D, Piek JP, Kane R, Palácio SG, Casella EB, et al. The relationship between motor skills, ADHD symptoms, and childhood body weight. Res Dev Disabil 2016;55:279–86. 10.1016/j.ridd.2016.05.005 27214681

[14] Cattuzzo MT, Dos Santos Henrique R, Ré AHN, Oliveira IS de, Melo BM, Sousa Moura M de, et al. Motor competence and health related physical fitness in youth: A systematic review. J Sci Med Sport 2016;19(2):123–9. 10.1016/j.jsams.2014.12.004 25554655

[15] Fogelholm M, Stigman S, Huisman T, Metsämuuronen J. Physical fitness in adolescents with normal weight and overweight. Scand J Med Sci Sports 2008;18(2):162–70. 10.1111/j.1600-0838.2007.00685.x 17490451

[16] Logan SW, Kipling Webster E, Getchell N, Pfeiffer KA, Robinson LE. Relationship between fundamental motor skill competence and physical activity during childhood and adolescence: a systematic review. Kinesiology Review 2015;4(4):416–26. 10.1123/kr.2013-0012

[17] Battaglia G, Alesi M, Tabacchi G, Palma A, Bellafiore M. The development of motor and pre-literacy skills by a physical education program in preschool children: a non-randomized pilot trial. Front Psychol 2018;9:2694. 10.3389/fpsyg.2018.02694 30687164 PMC6333915

[18] Holfelder B, Schott N. Relationship of fundamental movement skills and physical activity in children and adolescents: A systematic review. Psychol Sport Exerc 2014;15(4):382–91. 10.1016/j.psychsport.2014.03.005

[19] Okely AD, Booth ML, Patterson JW. Relationship of physical activity to fundamental movement skills among adolescents. Med Sci Sports Exerc 2001;33(11):1899–904. 10.1097/00005768-200111000-00015 11689741

[20] Lubans DR, Morgan PJ, Cliff DP, Barnett LM, Okely AD. Fundamental movement skills in children and adolescents: review of associated health benefits. Sports Med 2010;40(12):1019–35. 10.2165/11536850-000000000-00000 21058749

[21] Kalaja S, Jaakkola T, Liukkonen J, Watt A. Fundamental movement skills and motivational factors influencing engagement in physical activity. Percept Mot Skills 2010;111(1):115–28. 10.2466/06.10.25.PMS.111.4.115-128 21058593

[22] Fairclough SJ, Boddy LM, Hackett AF, Stratton G. Associations between children’s socioeconomic status, weight status, and sex, with screen-based sedentary behaviours and sport participation. Int J Pediatr Obes 2009;4(4):299–305. 10.3109/17477160902811215 19922045

[23] Poulain T, Vogel M, Sobek C, Hilbert A, Körner A, Kiess W. Associations between socio-economic status and child health: findings of a large german cohort study. Int J Environ Res Public Health 2019;16(5). 10.3390/ijerph16050677 30813530 PMC6427670

[24] Barnett LM, Lai SK, Veldman SLC, Hardy LL, Cliff DP, Morgan PJ, et al. Correlates of gross motor competence in children and adolescents: a systematic review and meta-analysis. Sports Med 2016;46(11):1663–88. 10.1007/s40279-016-0495-z 26894274 PMC5055571

[25] Quante M, Hesse M, Döhnert M, Fuchs M, Hirsch C, Sergeyev E, et al. The LIFE child study: a life course approach to disease and health. BMC Public Health 2012;12(1):1021. 10.1186/1471-2458-12-1021 23181778 PMC3533937

[26] Poulain T, Baber R, Vogel M, Pietzner D, Kirsten T, Jurkutat A, et al. The LIFE Child study: a population-based perinatal and pediatric cohort in Germany. Eur J Epidemiol 2017;32(2):145–58. 10.1007/s10654-016-0216-9 28144813

[27] World Medical Association. Declaration of Helsinki: ethical principles for medical research involving human subjects. JAMA 2013;310(20):2191–4. 10.1001/jama.2013.281053 24141714

[28] Kromeyer-Hauschild K, Wabitsch M, Kunze D, Geller F, Geiß HC, Hesse V, et al. Perzentile für den Body-mass-Index für das Kindes- und Jugendalter unter Heranziehung verschiedener deutscher Stichproben. Monatsschrift Kinderheilkunde 2001;149(8):807–18. 10.1007/s001120170107

[29] Wabitsch M, Kunze D. Konsensbasierte (S2) Leitlinie zur Diagnostik, Therapie und Prävention von Übergewicht und Adipositas im Kindes- und Jugendalter 2015.

[30] Finger JD, Mensink GBM, Banzer W, Lampert T, Tylleskär T. Physical activity, aerobic fitness and parental socio-economic position among adolescents: the German Health Interview and Examination Survey for Children and Adolescents 2003–2006 (KiGGS). Int J Behav Nutr Phys Act 2014;11(1):43. 10.1186/1479-5868-11-43 24656205 PMC3997963

[31] Poulain T, Vogel M, Ludwig J, Grafe N, Körner A, Kiess W. Reciprocal longitudinal associations between adolescents’ media consumption and psychological health. Academic Pediatrics 2018;19(1):109–17. 10.1016/j.acap.2018.08.009 30144525

[32] Lampert T, Sygusch R, Schlack R. Nutzung elektronischer Medien im Jugendalter. Ergebnisse des Kinder- und Jugendgesundheitssurveys (KiGGS). Bundesgesundheitsblatt Gesundheitsforschung Gesundheitsschutz 2007;50(5–6):643–52. 10.1007/s00103-007-0225-7 17514448

[33] Committee on Public Education. Children, adolescents, and television. Pediatrics 2001;107(2):423–6. 10.1542/peds.107.2.423 11158483

[34] Sauseng W, Sonnleitner A, Hofer N, Pansy J, Kiechl-Kohlendorfer U, Weiss S, et al. Empfehlungen zur Regulierung von Bildschirmzeiten im Kindes- und Jugendalter. Monatsschrift Kinderheilkunde 2017;165(3):254–6. 10.1007/s00112-016-0201-0

[35] Lampert T, Kroll LE, Müters S, Stolzenberg H. Messung des sozioökonomischen Status in der Studie „Gesundheit in Deutschland aktuell”(GEDA). Bundesgesundheitsblatt 2013;56(1):131–43. 10.1007/s00103-012-1583-3 23275948

[36] Rigby RA, Stasinopoulos DM. Generalized additive models for location, scale and shape (with discussion). J Royal Statistical Soc C 2005;54(3):507–54. 10.1111/j.1467-9876.2005.00510.x

[37] Krug S, Worth A, Finger JD, Damerow S, Manz K. Motorische Leistungsfähigkeit 4‑ bis 10‑jähriger Kinder in Deutschland: Ergebnisse aus KiGGS Welle 2 und Trends. Bundesgesundheitsblatt 2019;62(10):1242–52. 10.1007/s00103-019-03016-7 31529188

[38] Tomkinson GR, Carver KD, Atkinson F, Daniell ND, Lewis LK, Fitzgerald JS, et al. European normative values for physical fitness in children and adolescents aged 9–17 years: results from 2 779 165 Eurofit performances representing 30 countries. Br J Sports Med 2018;52(22):1445–14563. 10.1136/bjsports-2017-098253 29191931

[39] Xu Y, Mei M, Wang H, Yan Q, He G. Association between Weight Status and Physical Fitness in Chinese Mainland Children and Adolescents: A Cross-Sectional Study. Int J Environ Res Public Health 2020;17(7). 10.3390/ijerph17072468 32260379 PMC7177678

[40] Ervin RB, Wang C-Y, Fryar CD, Miller IM, Ogden CL. Measures of muscular strength in U.S. children and adolescents, 2012. NCHS Data Brief 2013(139):1–8. 24331231

[41] Weihrauch-Blüher S, Wiegand S. Risk factors and implications of childhood obesity. Curr Obes Rep 2018;7(4):254–9. 10.1007/s13679-018-0320-0 30315490

[42] Morgan PJ, Barnett LM, Cliff DP, Okely AD, Scott HA, Cohen KE, et al. Fundamental movement skill interventions in youth: a systematic review and meta-analysis. Pediatrics 2013;132(5):e1361–83. 10.1542/peds.2013-1167 24167179

[43] Lloyd RS, Cronin JB, Faigenbaum AD, Haff GG, Howard R, Kraemer WJ, et al. National strength and conditioning association position statement on long-term athletic development. J Strength Cond Res 2016;30(6):1491–509. 10.1519/JSC.0000000000001387 26933920

[44] Rose B, Larkin D, Berger BG. The importance of motor coordination for children’s motivational orientations in sport. Adapted Physical Activity Quarterly 1998;15(4):316–27. 10.1123/apaq.15.4.316

[45] an de Meester, Maes J, Stodden D, Cardon G, Goodway J, Lenoir M, et al. Identifying profiles of actual and perceived motor competence among adolescents: associations with motivation, physical activity, and sports participation. J Sports Sci 2016;34(21):2027–37. 10.1080/02640414.2016.1149608 26928601

[46] Tremblay MS, Leblanc AG, Kho ME, Saunders TJ, Larouche R, Colley RC, et al. Systematic review of sedentary behaviour and health indicators in school-aged children and youth. Int J Behav Nutr Phys Act 2011;8:98. 10.1186/1479-5868-8-98 21936895 PMC3186735

[47] Jones MA, Hitchen PJ, Stratton G. The importance of considering biological maturity when assessing physical fitness measures in girls and boys aged 10 to 16 years. Ann Hum Biol 2000;27(1):57–65. 10.1080/030144600282389 10673141

[48] Guimarães E, Ramos A, Janeira MA, Baxter-Jones ADG, Maia J. How Does Biological Maturation and Training Experience Impact the Physical and Technical Performance of 11–14-Year-Old Male Basketball Players? Sports 2019;7(12):243. 10.3390/sports7120243 31816896 PMC6956237

