# Supplementary figures and images for: Motor skills in relation to body-mass index, physical activity, TV-watching, and socioeconomic status in German four-to-17-year-old children
Source: PLoS One. 2021 May 17;16(5):e0251738. doi: 10.1371/journal.pone.0251738 (PMC8128247; doi:10.1371/journal.pone.0251738)

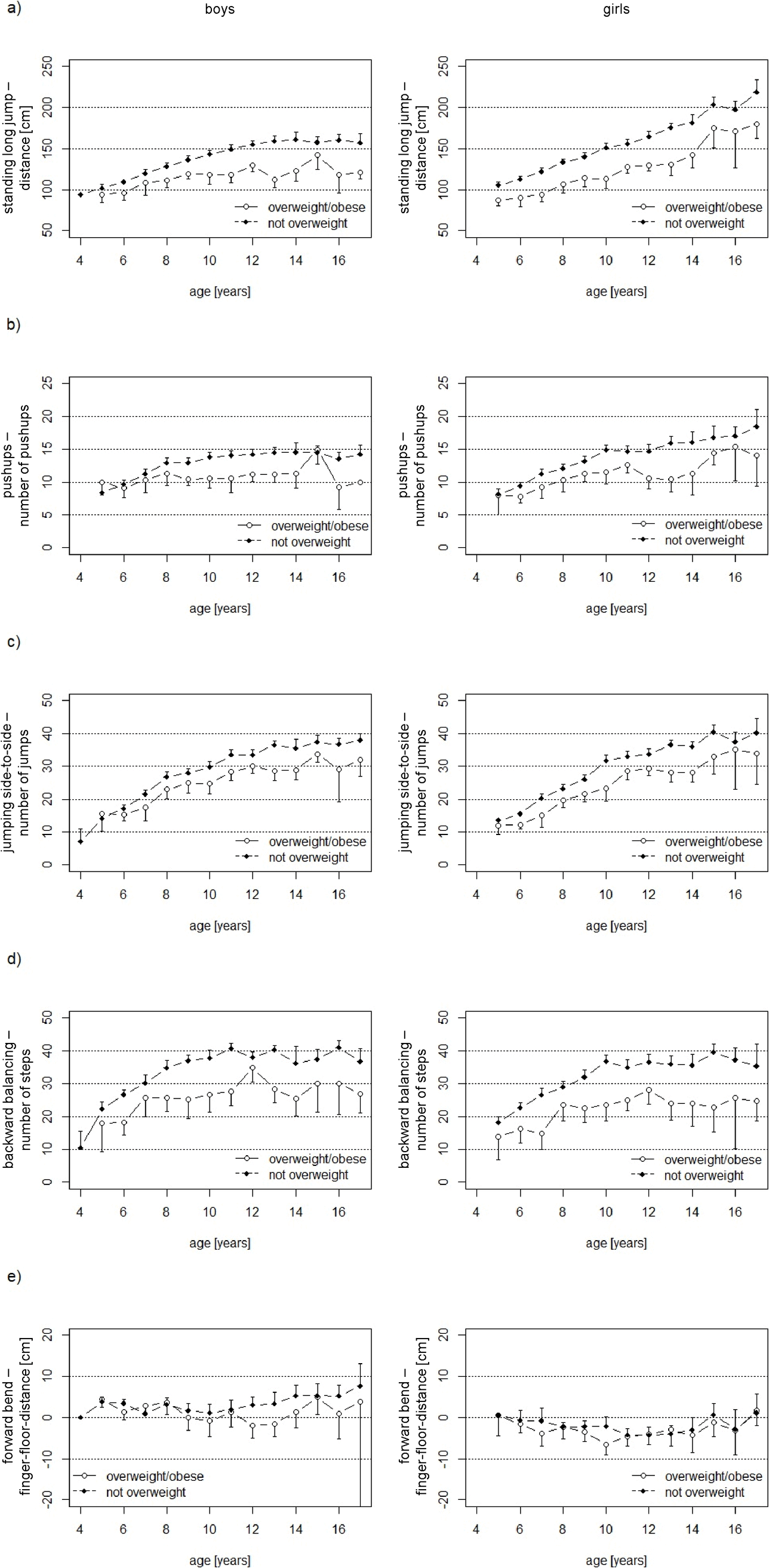

Supplement: S1 Fig — Raw scores in the different tasks of the motoric test by age, weight status, and gender (boys on the left, girls on the right). (a) best distance of two tries doing a standing long jump. (b) number of pushups during 40s. (c) mean number of side to side jumps performed in a 15s interval. (d) Number of steps taken on six tries balancing backwards on three beams of different widths. (e) best distance (cm) between fingertips and bench reached in two tries (negative numbers indicate bench level was not reached, positive numbers indicate bench level was exceeded). Error bars indicate 95% confidence intervals. (TIF) [file pone.0251738.s001.tif]

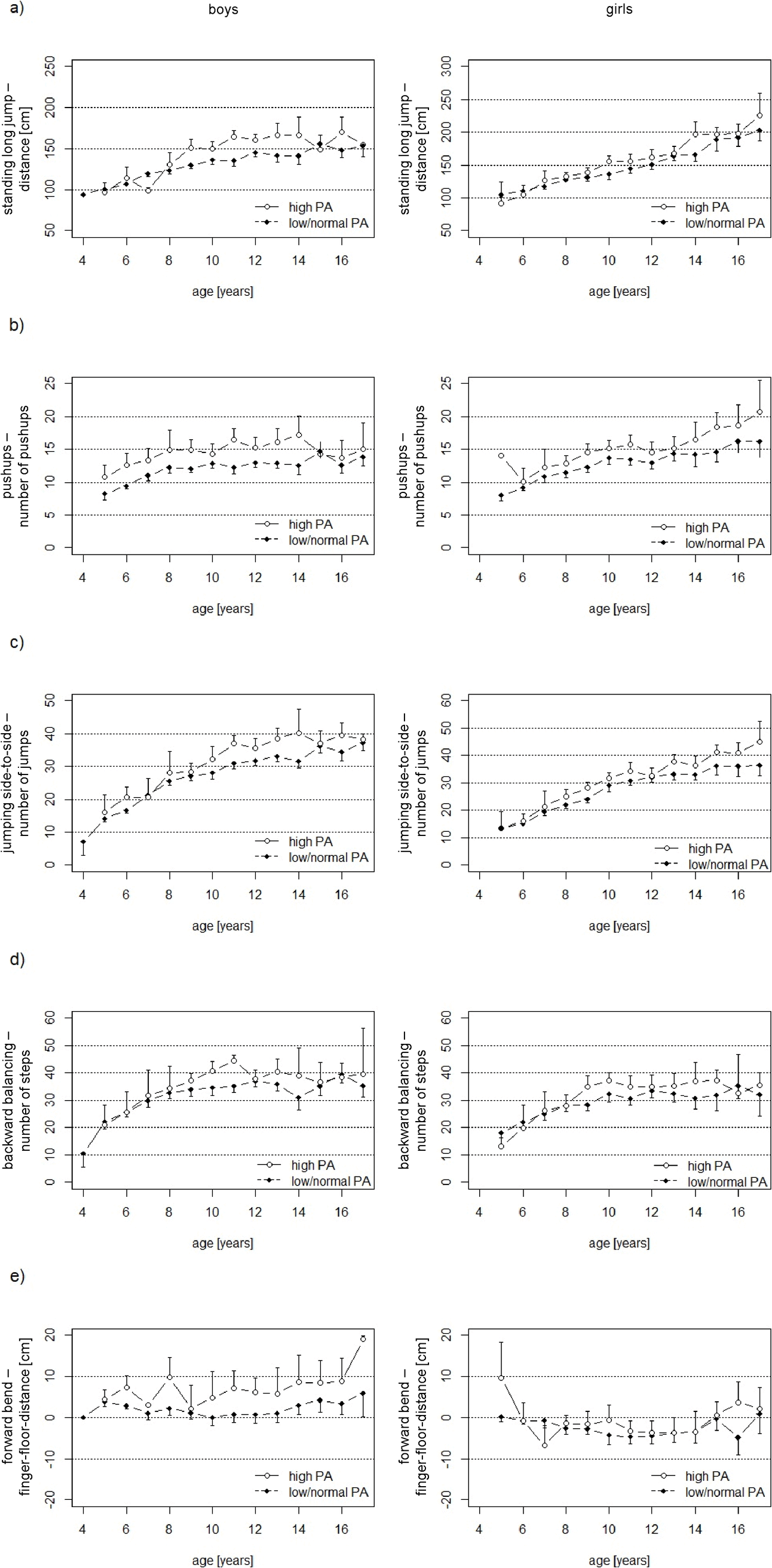

Supplement: S2 Fig — Raw scores in the different tasks of the motoric test by age, level of PA, and gender (boys on the left, girls on the right). (a) best distance of two tries doing a standing long jump. (b) number of pushups during 40s. (c) mean number of side to side jumps performed in a 15s interval. (d) Number of steps taken on six tries balancing backwards on three beams of different widths. (e) best distance (cm) between fingertips and bench reached in two tries (negative numbers indicate bench level was not reached, positive numbers indicate bench level was exceeded). Error bars indicate 95% confidence intervals. (TIF) [file pone.0251738.s002.tif]

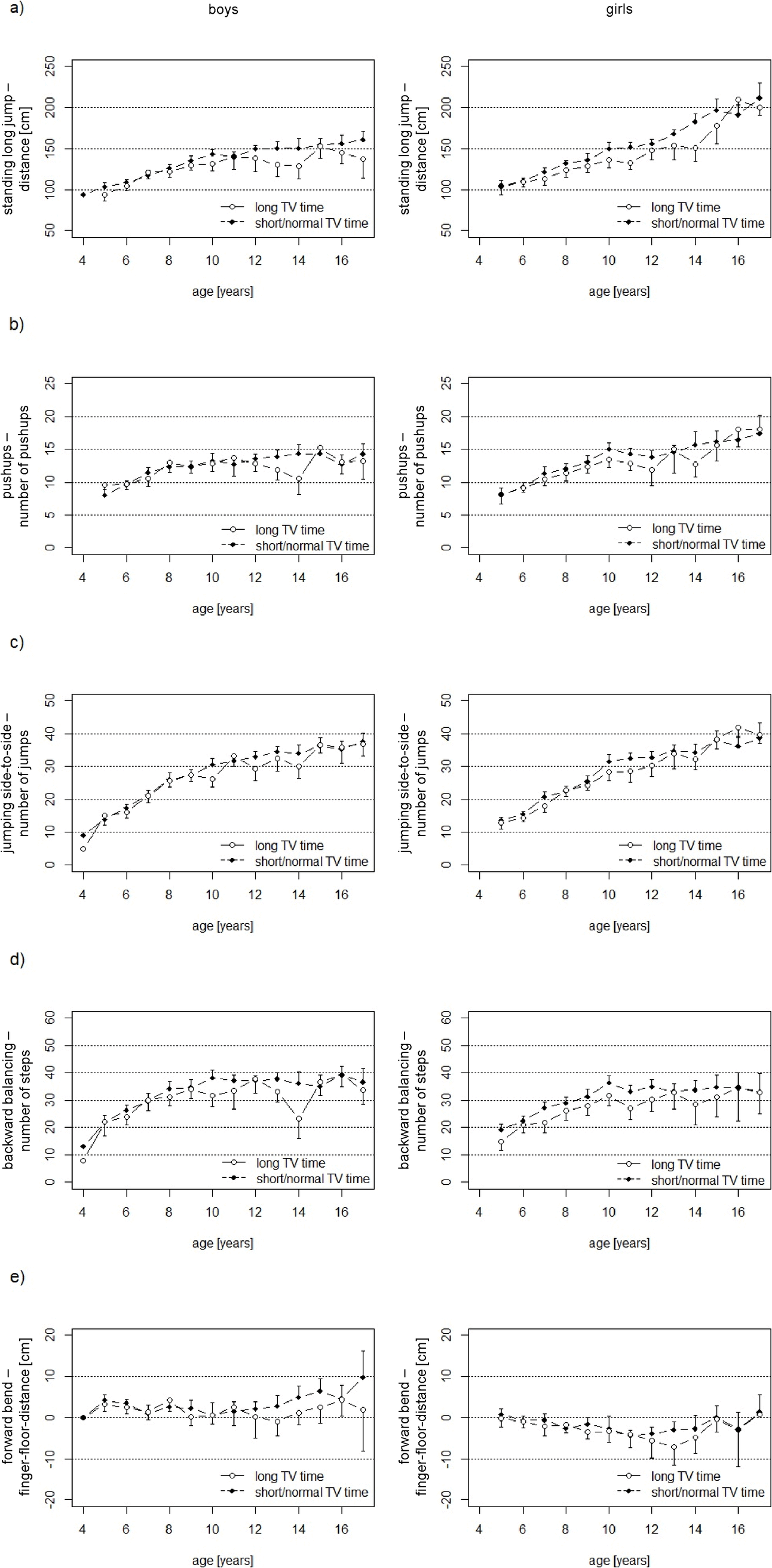

Supplement: S3 Fig — Raw scores in the different tasks of the motoric test by age, TV time, and gender (boys on the left, girls on the right). (a) best distance of two tries doing a standing long jump. (b) number of pushups during 40s. (c) mean number of side to side jumps performed in a 15s interval. (d) Number of steps taken on six tries balancing backwards on three beams of different widths. (e) best distance (cm) between fingertips and bench reached in two tries (negative numbers indicate bench level was not reached, positive numbers indicate bench level was exceeded). Error bars indicate 95% confidence intervals. (TIF) [file pone.0251738.s003.tif]

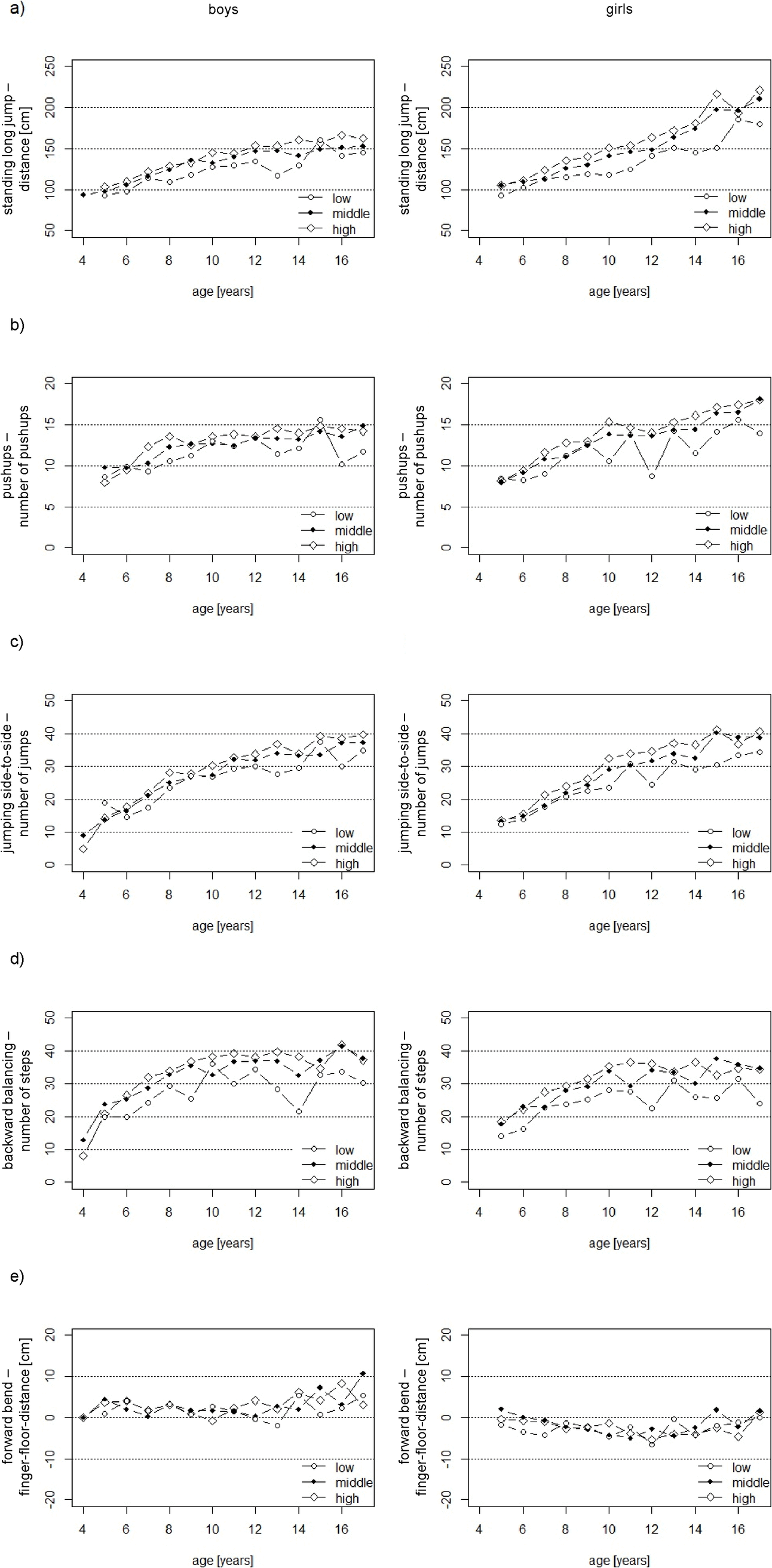

Supplement: S4 Fig — Raw scores in the different tasks of the motoric test by age, SES, and gender (boys on the left, girls on the right). (a) best distance of two tries doing a standing long jump. (b) number of pushups during 40s. (c) mean number of side to side jumps performed in a 15s interval. (d) Number of steps taken on six tries balancing backwards on three beams of different widths. (e) best distance (cm) between fingertips and bench reached in two tries (negative numbers indicate bench level was not reached, positive numbers indicate bench level was exceeded). Error bars indicate 95% confidence intervals. (TIF) [file pone.0251738.s004.tif]
