# Supplementary material for: Motor skills in relation to body-mass index, physical activity, TV-watching, and socioeconomic status in German four-to-17-year-old children
Source: PLoS One. 2021 May 17;16(5):e0251738. doi: 10.1371/journal.pone.0251738 (PMC8128247; doi:10.1371/journal.pone.0251738)
Supplement: S1 Table — (DOCX) [file pone.0251738.s005.docx]

**S1 Table. Questions of the leisure-time-behavior questionnaires**

Article: Motor skills in relation to body-mass index, physical activity, TV-watching, and socioeconomic status in German four-to-17-year-old children

Authors: Siegfried Möller, Tanja Poulain, Antje Körner, Christof Meigen, Anne Jurkutat, Mandy Vogel, Sven Wessala, Andreas Hiemisch, Nico Grafe and Wieland Kiess

| German (original) | English (translation) |
| --- | --- |
| **Leisure time behavior, answered by the participants (used for 11-year-old and older participants)** | |
| 1. Wie häufig treibst du Sport in einem Verein?  **Antwortmöglichkeiten:** Nie, Seltener, 1-2 mal pro Woche, 3-5 mal pro Woche, Fast jeden Tag | 1. Question: How often do you participate in organized sports (at a sports club)?  **Answer options:** Never, Less often, 1 to 2 times a week, 3 to 5 times a week, Almost every day |
| 2. Wie lange beschäftigst du dich durchschnittlich pro Tag mit folgenden Dingen? – Fernsehen/Video  **Antwortmöglichkeiten:** Gar nicht, Ungefähr 30 Minuten pro Tag, Ungefähr 1-2 Stunden pro Tag, Ungefähr 3-4 Stunden pro Tag, Mehr als 4 Stunden pro Tag | 2. Question: On average, how long do you engage in the following activities per day? – TV/Video  **Answer options:** Not at all, About 30 minutes per day, About 1-2 hours per day, About 3-4 hours per day, More than 4 hours per day |
| **Leisure time behavior, answered by the participants’ parents (used for 10-year-old and younger participants)** | |
| 1. Wie häufig treibt Ihr Kind Sport in einem Verein?  **Antwortmöglichkeiten:** Nie, Seltener, 1-2 mal pro Woche, 3-5 mal pro Woche, Fast jeden Tag, Nie | 1. How often does your child participate in organized sports (at a sports club)?  **Answer options:** Never, Less often, 1 to 2 times a week, 3 to 5 times a week, Almost every day |
| 2. Wie lange beschäftigt sich Ihr Kind pro Tag mit Fernsehen/Video?  **Antwortmöglichkeiten:** Gar nicht, Ungefähr 30 Minuten pro Tag, Ungefähr 1-2 Stunden pro Tag, Ungefähr 3-4 Stunden pro Tag, Mehr als 4 Stunden pro Tag | 2. How much time per day does your child spend watching TV/videos?  **Answer options:** Not at all, About 30 minutes per day, About 1-2 hours per day, About 3-4 hours per day, More than 4 hours per day |
